# Five-year change of prevalence and risk factors for infection and mortality of carbapenem-resistant *Klebsiella pneumoniae* bloodstream infection in a tertiary hospital in North China

**DOI:** 10.1186/s13756-020-00728-3

**Published:** 2020-06-01

**Authors:** Yuanyuan Li, Jihong Li, Tong Hu, Jia Hu, Ning Song, Yu Zhang, Yuan Chen

**Affiliations:** 1 Department of Infectious Diseases, The Second Hospital of Hebei Medical University, No. 215 Heping Western Road, Xinhua District, Shijiazhuang, 050000 China; 2 Department of Laboratory Medicine, The Second Hospital of Hebei Medical University, No. 215 Heping Western Road, Shijiazhuang, 050000 China; 3 Hebei Medical University Fourth Affiliated Hospital and Hebei Provincial Tumor Hospital, No.12 Jiankang Road, Shijiazhuang, 050000 China; 4 Department of Pediatrics, The Second Hospital of Hebei Medical University, No. 215 Heping Western Road, Shijiazhuang, 050000 China

**Keywords:** Carbapenem resistant *Klebsiella pneumoniae* (CRKP), Bloodstream infection, Prevalence, Mortality, Risk factors

## Abstract

**Background:**

There are few studies focused on carbapenem-resistant *Klebsiella pneumoniae* (CRKP) bloodstream infection (BSI). The aim of this study is to identify the prevalence and risk factors for infection and mortality of CRKP BSI.

**Methods:**

Susceptibility of *Klebsiella pneumoniae* (KP) isolated from blood samples and the proportion of CRKP were recorded annually. One hundred sixty-four patients with CRKP and 328 with carbapenem-susceptible *Klebsiella pneumoniae* (CSKP) BSI were categorized as the case group and control group to identify risk factors for CRKP infection and mortality by univariable analysis and multivariable logistic-regression analysis.

**Results:**

The proportion and mortality of CRKP BSI increased significantly, with the percentage of KP in BSI increasing from 7 to 12% from 2014 to 2019 with a concomitant resistance to meropenem increasing from 16.7 to 41.8%. Compared with CSKP group, patients in CRKP group had longer hospitalization time before bacteremia (median 14 vs 4, *P* < 0.001) and longer total hospitalization time (median 31 vs 19, *P* < 0.001). The proportion of admission to ICU was higher (70.7% vs 17.7%, P < 0.001), and APACHE II score was higher (median 12 vs 8, P < 0.001). The mortality in CRKP group was 43.9% (72/164), while 14.9% (49/328) in CSKP group (*p* < 0.001). KP detection in other sites(*P* = 0.036, OR 1.964), blood purification(*P* = 0.018, OR 3.326), bronchoscopy(*P* = 0.011, OR 5.423), surgery (*P* = 0.001, OR 3.084), carbapenem use(P = 0.001, OR 3.395), tigecycline use(*P* = 0.006, OR 4.595) were independent risk factors for CRKP BSI. Previous hospitalization (*P* = 0.048, OR 2.755), long hospitalization (*P* = 0.003, OR 1.035), bone marrow puncture (*P* = 0.037, OR3.856), use of β-lactamase inhibitor (*P* = 0.005, OR 3.890) were independent risk factors for mortality in CRKP BSI.

**Conclusion:**

The prevalence and mortality of CRKP BSI are still increasing. Timely treatment of KP infection in other site, strengthening the hospital infection control of blood purification, bronchoscopy and surgery, control the use of carbapenem and tigecycline, may help to prevent CRKP BSI. More preventative hospital resources are needed for severely ill patients with prolonged hospitalizations and intensive care.

## Introduction

Carbapenems are the most effective and reliable β-lactams for the treatment of severe infections caused by multidrug resistant Enterobacteriaceae [1, 2]. In the past 10 years, carbapenems have been regarded as the last line of defense in the treatment of drug-resistant gram-negative bacterial infections. However, with the extensive use of carbapenems, many bacteria resistant to carbapenems have appeared, carbapenem resistant *Klebsiella pneumoniae* (CRKP) is one of which.

CRKP was first discovered in North Carolina in 1996, and now it has become the most common type of carbapenem resistant *Enterobacteriaceae* (CRE) in the United States [3]. At the same time, CRKP is also prevalent in Israel [4], Europe [5] and some South American countries [6, 7]. Carbapenem resistance among *Klebsiella pneumoniae* (KP) in the United States was as high as 12% of all isolates in 2009–2010 [8], while it was less than 1% in 2000 [3]. In Europe, It was 7.2% in 2017, Greece was the highest, 64.7% [9].

According to the data of China Antimicrobial Surveillance Network (CHINET) in 2018 [10], the proportion of KP in CRE strains is 73.5% and the resistance rates of KP to imipenem and meropenem were 26.3 and 25% respectively. The top five provinces with the highest prevalence of CRKP in China *were* Henan Province (61.8%), Shanxi Province (58.3%), Beijing City (55.7%), Zhejiang Province (53.3%) and Hebei Province (38%). However, CHINET only included data of KP from all parts of the body, the data of bloodstream infection (BSI) was not available.

Many studies have shown that CRKP significantly prolongs hospital stays and increases mortality compared to carbapenem-susceptible *Klebsiella pneumoniae* (CSKP) [11, 12]. Among the infections caused by CRKP in many sites, BSI is the most important type of infection with a high mortality rate [13]. A case-control study showed that the mortality rate of CRKP BSI was as high as 71.9%, which is much higher than the 21.9% of CRKP infection in other sites [14]. At present, there are many studies on the risk factors of CRKP infection, but most of them do not distinguish the infection sites. In this study, we will focus on BSI to analyze prevalence, mortality and risk factors for CRKP infection and mortality.

## Materials and methods

### Study design

Susceptibility of KP isolated from blood samples and the proportion of CRKP were recorded annually. To identify the risk factors for CRKP infections, we conducted a retrospective case-control study at the second hospital of Hebei Medical University, which is a grade III, class A university affiliated hospital in Shijiazhuang, Hebei province in North China with 2800 beds. All the adult inpatients (age ≥ 18 years) with positive blood cultures of KP, both CRKP and CSKP, who met diagnostic criteria of BSI according to the CDC / NHSN standard [15], were selected from the medical records in the hospital’s computerized microbiology laboratory database, dated between January 1, 2014 and June 30, 2019. The first positive sample of each patient was analyzed in the study. The mortality of KP, CRKP and CSKP BSI in each year and the total mortality in 5 years were calculated and analyzed. The prevalence of CRKP means the proportion of CRKP in KP strains.

### Antimicrobial susceptibility testing

BacT / Alert3D automatic blood culture instrument is used for blood culture. Strain identification was performed with the Vitek2-compact automated microbiology system. Antimicrobial susceptibilities were determined by the VITEK system or the disk diffusion method. The results were interpreted according to the criteria recommended by the Clinical Laboratory Standards Institute (CLSIM100-S28), in which the US FDA standard was adopted for tigecycline test. CRKP was defined as an isolate with ertapenem (MICs ≥2 μg/ml), or imipenem and/or meropenem (MICs ≥4 μg/ml). The KP isolates susceptible to ertapenem, imipenem, and meropenem were considered as CSKP [16]. *Escherichia coli* ATCC 25922, and *Pseudomonas aeruginosa* ATCC 27853 were used as internal quality control.

### Data collection

The following data was collected from medical records of each patient: ① Demographic characteristics: gender, age, length of stay, etc.; ② Comorbidities and underlying diseases: cardiovascular, pulmonary, renal disease, diabetes mellitus, solid tumor, hematological malignancy and immunocompromised; ③ Disease severity: acute physiology and chronic health status scoring system II (APACHE II)score; ④ Hospitalization and treatment before KP were isolated: admission to intensive care unit (ICU), previous hospitalization (≤3 months), special treatment (Corticosteroids, radiotherapy / chemotherapy), previous surgery (within 1 month) and recent invasive procedures (nasogastric or urinary catheter, central venous catheter (CVC), peripheral arterial catheter, blood purification (including continuous renal replacement therapy (CRRT), bedside hemofiltration, hemodialysis, plasmapheresis), tracheal cannula, tracheotomy, non-invasive mechanical ventilation, bone marrow puncture, bronchoscopy, parenteral nutrition, etc. a total of 17 items), use of antibiotics. ⑤ KP detection in other sites and death during the current admission.

### Statistical analysis

SPSS version 22.0 was used to perform all the statistical analysis. Continuous variables were described as mean ± standard deviation (SD), which was compared using the Student’s t-test, if their distributions were not normal, they were described as median (quartiles) and were compared with the Mann-Whitney U test. Categorical variables were compared with a Pearson’s Chi-square test or a Chi-square test with a continuity correction if the frequency is < 5. Variables with P<0.05 in the univariable analysis above were included in multivariable logistic regression model. We calculated the odds ratios (ORs) and the 95% confidence intervals (CIs) for each variable. In multivariable logistic regression, the risk factors whose P<0.05 and OR > 1 were considered as independent risk factors. We used Hosmer-Lemeshow test to test the goodness of fit for logistic regression model and the logistic model is reliable when P>0.05 in this test.

## Results

### Trends in prevalence and mortality of CRKP BSI over the past 5 years

**Fig. 1:**
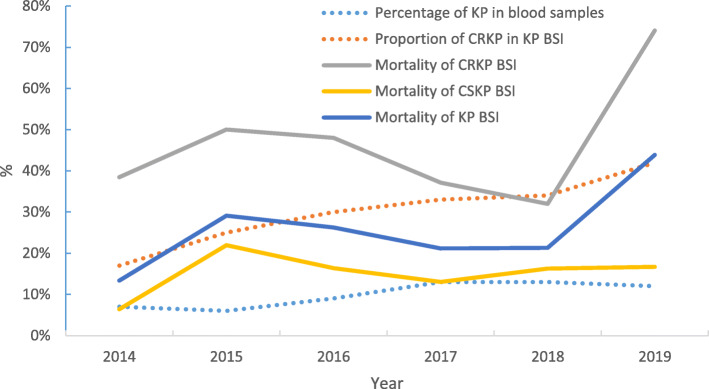
Trends in prevalence and mortality of CRKP BSI over the past 5 years. The prevalence of KP BSI and the proportion of CRKP has been increasing year by year and the mortality of KP BSI also increased. The mortality of CRKP BSI is higher than that of CSKP. KP: *Klebsiella pneumoniae*; CRKP: Carbapenem-resistant KP; CSKP: Carbapenem-susceptible KP; BSI: bloodstream infection

A total of 5545 strains were cultured and identified in the blood samples in our hospital, including 551 KP strains. The percentage of KP in blood sample from 2014 to the first half of 2019 were 7, 6, 9, 13, 13 and 12% respectively, which rose fastest from 2015 to 2017, then tended to be stable from 2017 to the first half of 2019. The percentage of CRKP in KP BSI increased annually from 17% in 2014 to 42% in the first half of 2019, as shown in Fig. 1.

Over the past 5 years, the drug resistance rate of KP detected in blood samples to all drugs except tigecycline has been increasing, especially to carbapenems (details are shown in Table 1).

**Table 1:** Susceptibility of *Klebsiella pneumoniae* to antimicrobial agents from 2014 to 2019

Antimicrobial agents	2014(*n* = 72)		2015(*n* = 59)		2016(*n* = 88)		2017(*n* = 115)		2018(*n* = 150)		2019(*n* = 67)	
	R(%)	S(%)	R(%)	S(%)	R(%)	S(%)	R(%)	S(%)	R(%)	S(%)	R(%)	S(%)
Ampicillin	84.7	2.8	83.1	0	84.1	0	93	0	94.4	0	95.5	0
Ampicillin / sulbactam	43.1	50	61	35.6	46.6	52.3	64.3	34.8	65.3	29.3	63.6	33.3
Piperacillin	40.3	54.2	61	35.6	45.3	52.3	61.1	34.5	66	31.3	67.2	32.8
Piperacillin / tazobactam	22.2	75	30.5	66.1	30.2	68.6	37.2	59.3	35.6	60.4	43.3	55.2
Cefuroxime	40.3	54.2	59.3	40.7	45.5	52.3	62.6	36.5	64.7	32	67.2	31.3
Cefatriaxone	40.3	59.7	59.3	40.7	44.3	55.7	62.6	37.4	64.7	35.3	65.7	34.3
Ceftazidime	29.2	70.8	39	59.3	35.2	64.8	50.4	46.1	45.3	50.7	52.2	46.3
Cefepime	25	73.6	28.8	62.7	35.2	63.6	50.4	47.8	42	53.3	51.5	47
Cefotetan	18.1	79.2	23.7	76.3	21.6	78.4	31.3	66.1	28.7	70	38.8	59.7
Cefoperazone / sulbactam	27.6	69	22.2	61.1	24.7	71.4	38.5	51.9	32.6	57.2	45.2	51.6
Aztreonam	31.9	68.1	37.3	61	38.6	61.4	56.5	43.5	51.3	48.7	61.2	38.8
Levofloxacin	28.1	71.9	33.9	59.3	33	67	50.4	49.6	38.7	57.3	55.2	43.3
Ciprofloxacin	29.2	68.1	37.3	59.3	35.2	62.5	53	43.5	46.7	50.7	56.7	43.3
Gentamicin	34.7	34.7	42.4	55.9	30.7	68.2	44.3	55.7	42	55.3	44.8	50.7
Tobramycin	13.9	72.2	25.4	52.5	30.7	61.4	41.7	46.1	24	54.7	38.8	53.7
Imipenem	22.2	76.4	25.4	71.2	29.5	69.3	34.8	64.3	34	65.3	43.3	53.7
Meropenem	16.7	83.3	25.4	74.6	30.2	68.6	33.6	65.5	34.2	65.8	41.8	58.2
Amikacin	8.3	91.7	16.9	81.4	14	86	25.2	73.9	12.8	87.2	28.4	71.6
SMZ-TMP	36.1	63.9	45.8	54.2	36.4	63.6	52.2	47.8	47.7	52.3	40.3	59.7
Tigecycline									0	65.9	0	67

*R* Resistance, *S* susceptible

Four hundred ninety-two patients who met the criteria for BSI included 164 (33.3%) CRKP cases and 328 (66.7%) CSKP cases, among which 121 patients (24.6%) died during the current admission. The mortality in CRKP group was 43.9% (72/164), while 14.9% (49/328) in CSKP group. The difference was statistically significant (P<0.001). The mortality of KP BSI has increased from 14% in 2014 to 44% in the first half of 2019. The mortality of CRKP BSI and CSKP BSI increased from 38 and 6% in 2014 to 74 and 17% in the first half of 2019, respectively. Details were shown in Fig. 1.

**Fig. 2:**
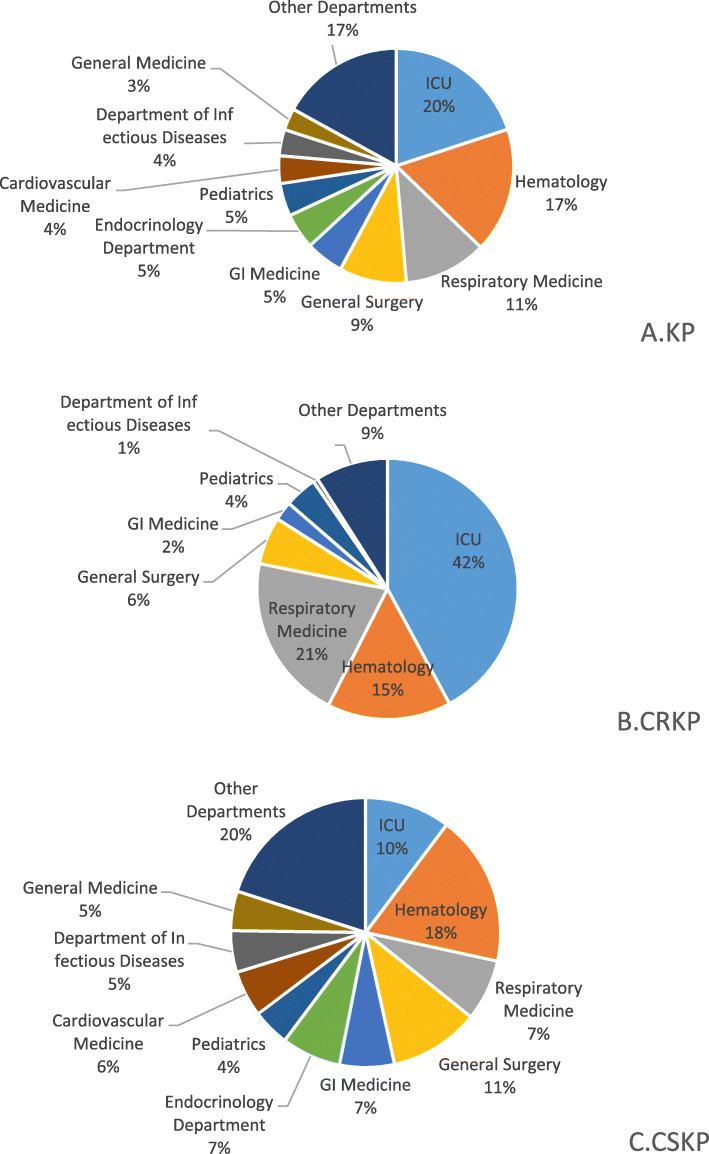
Distribution of KP, CRKP and CSKP in various Departments in the last 5 years. **a**, **b** and **c** showed the distribution of KP, CRKP and CSKP in various departments in the last 5 years, respectively. Half of the CRKP was distributed in ICU, while the distribution of CSKP in each department was relatively discrete. KP: *Klebsiella pneumoniae*; CRKP: Carbapenem-resistant KP; CSKP: Carbapenem-susceptible KP; ICU: Intensive Care Unit

KP BSI was mainly distributed in Intensive Care Unit (ICU), Hematology Department, Respiratory Medicine and General Surgery Department (20, 17, 11 and 9% respectively, as shown in Fig. 2a). Nearly half of the CRKP was distributed in ICU (42%), followed by Respiratory Medicine (21%) and Hematology Department (15%). Details were shown in Fig. 2b. In 2014, 83% of the KP detected in blood from ICU was CRKP, which decreased and stabilized between 50 to 63% in the following years. The proportion of CRKP in KP rose to 42% in the first half of 2019 from 8% in 2014 in Hematology Department. Hematology Department and ICU account for 40% of CRKP BSI in 2014, which rose to 64% in the first half of 2019. CSKP was mainly detected from Hematology, General surgery and ICU (18, 11 and 10% respectively). Details were shown in Fig. 2c.

### Risk factors for CRKP BSI

Compared with CSKP group, patients in CRKP group had longer hospitalization time before bacteremia (median 14 vs 4, *P* < 0.001) and longer total hospitalization time (median 31 vs 19, *P* < 0.001). The proportion of admission to ICU was higher (70.7% vs 17.7%, P < 0.001), and APACHE II score was higher (median 12 vs 8, P < 0.001). In terms of underlying diseases, the proportion of hypertension, chronic obstructive pulmonary disease (COPD) and kidney disease in CRKP group was higher (43.9% vs 34.1, 8.5% vs 2.7, 13.4% vs 6.1%, *P* < 0.05), and that of diabetes was higher in CSKP group (20.7% vs 37.8%, *P* < 0.001).

In addition to blood samples, KP could be detected in samples from other sites in 186(37.8%) patients. The proportion of KP detection in other sites in CRKP group was higher than that in CSKP group (65.9% VS 23.8%, P < 0.001). In addition to blood samples, KP could be detected in one site in 63 (38.4%) patients in CRKP group, while 69(21%) in CSKP group. And KP could be detected in two or more other sites in 45 (27.4%) patients in CRKP group, while 9(2.7%) in CSKP group. Details were shown in Table 2.

**Table 2:** KP detection in samples from other sites

KP detection in samples from other sites	KP (*n* = 492)	CRKP (*n* = 164)	CSKP (*n* = 328)
KP detection in samples from other sites	186(37.8%)	108(65.9%)	78(23.8%)
1 site	132	63	69
Sputum	75	49	26
Urine	15	1	14
Drainage fluid of liver abscess	13	0	13
Pleural/peritoneal effusion	11	8	3
Skin and soft tissue	6	3	3
Bile	6	1	5
Cerebrospinal fluid	2	0	2
Catheter tip	2	1	1
Stool	2	0	2
2 sites	45	36	9
Sputum+ Pleural/peritoneal effusion	18	16	2
Sputum+ Urine	9	8	1
Sputum+ Catheter tip	6	2	4
Sputum+ Skin and soft tissue	4	4	0
Sputum+ Cerebrospinal fluid	2	2	0
Sputum+ Drainage fluid of liver abscess	2	0	2
Sputum+ Pleural/peritoneal effusion	2	2	0
Sputum+ Stool	1	1	0
Stool+ Skin and soft tissue	1	1	0
3 sites	7	7	0
Sputum+ Urine+ Pleural/peritoneal effusion	4	4	0
Sputum+ Skin and soft tissue+ Catheter tip	1	1	0
Sputum+ Urine+ Bile	1	1	0
Sputum+ Urine+ Catheter tip	1	1	0
4 sites	2	2	0
Sputum+ Skin and soft tissue + Catheter tip + Pleural/peritoneal effusion	2	2	0

*KP Klebsiella pneumoniae*, *CRKP* Carbapenem-resistant KP, *CSKP* Carbapenem-susceptible KP

In the total 492 patients, in addition to blood samples, KP could be detected in samples from other sites in 186(37.8%) patients, among which, 108(65.9%) were in CRKP group, which was significantly higher than 78(23.8%) in CSKP group (P < 0.001); In addition to blood samples, KP could be detected in one site in 63 (38.4%) patients in CRKP group, while 69(21%) in CSKP group; KP could be detected in two or more other sites in 45 (27.4%) patients in CRKP group, while 9(2.7%) in CSKP group

Univariate analysis showed that enema, indwelling nasogastric catheter, urinary catheter, CVC, peripheral arterial catheter, blood purification, tracheal cannula, tracheotomy, non-invasive ventilation, bronchoscopy, sputum aspiration, thoracentesis, abdominocentesis, lumbar puncture, previous surgery, enteral and parenteral nutrition, previous use of any antibiotics, carbapenems, glycopeptides, quinolones, β-lactamase inhibitors, linezolid and tigecycline were associated with CRKP BSI. Details were shown in Table 3.

**Table 3:** Comparison of clinical characteristics between CRKP group and CSKP group

Items	CRKP (*n* = 164)	CSKP (*n* = 328)	Z /χ^2^	*P* value
Male	110(67.1%)	195(59.5%)	2.696	0.101
Age (years)	56(29)	59(19)	−0.103	0.918
Previous hospitalization	121(73.8%)	214(65.2%)	3.667	0.056
Hospital stay (days)	31(30)	19(19)	−5.973	**<0.001**
Hospital stay before bacteremia (days)	14(20)	4(12)	−8.556	**<0.001**
APACHE II score	12(6)	8(5)	−9.025	**<0.001**
ICU admission	116(70.7%)	58(17.7%)	134.604	**<0.001**
Use of systemic steroids	46(28%)	68(20.7%)	3.288	0.070
Chemotherapy/Radiotherapy	30(18.3%)	72(22%)	0.890	0.345
Use of immunosuppressant	34(20.7%)	79(24.1%)	0.695	0.404
Hypertension	72(43.9%)	112(34.1%)	4.445	**0.035**
Coronary heart disease	37(22.6%)	67(20.4%)	0.299	0.585
Diabetes mellitus	34(20.7%)	124(37.8%)	14.619	**<0.001**
COPD	14(8.5%)	9(2.7%)	8.233	**0.004**
Renal diseases	22(13.4%)	20(6.1%)	7.497	**0.006**
Autoimmune diseases	7(4.3%)	10(3.0%)	0.487	0.485
Solid tumor	19(11.6%)	45(13.7%)	0.440	0.507
Hematologic malignancy	24(14.6%)	55(16.8%)	0.369	0.543
Enema	14(8.5%)	9(2.7%)	8.233	**0.004**
Nasogastric catheter	117(71.3%)	76(23.2%)	106.419	**<0.001**
Urinary catheter	126(76.8%)	95(29%)	101.245	**<0.001**
CVC	142(86.6%)	126(38.4%)	102.298	**<0.001**
Peripheral arterial catheter	34(20.7%)	17(5.2%)	28.499	**<0.001**
Blood purification	36(22.0%)	11(3.4%)	43.766	**<0.001**
Tracheal cannula	94(57.3%)	39(11.9%)	114.383	**<0.001**
Tracheostomy	35(21.3%)	21(6.4%)	24.191	**<0.001**
Non-invasive ventilation	22(13.4%)	15(4.6%)	12.215	**<0.001**
Gastroscopy	6(3.7%)	6(1.8%)	0.865	0.352
Colonoscopy	2(1.2%)	1(0.3%)	0.377	0.539
Bronchoscopy	35(21.3%)	4(1.2%)	60.654	**<0.001**
Sputum suction	102(62.2%)	48(14.6%)	116.699	**<0.001**
Thoracentesis	37(22.6%)	9(2.7%)	50.660	**<0.001**
Abdominocentesis	19(11.6%)	5(1.5%)	23.851	**<0.001**
Bone marrow puncture	26(15.9%)	56(17.1%)	0.117	0.732
Lumbar puncture	24(14.6%)	24(7.3%)	6.649	**0.01**
Previous surgery	87(53.0%)	82(25.0%)	38.144	**<0.001**
Abdominal surgery	50(30.5%)	88(26.8%)	0.725	0.394
Enteral nutrition	80(48.8%)	44(13.4%)	72.541	**<0.001**
Parenteral nutrition Previous use of antibiotics	95(57.9%)	73(22.3%)	61.866	**<0.001**
Any antibiotics	158(96.3%)	229(69.8%)	45.822	**<0.001**
Carbapenems	96(58.5%)	44(13.4%)	109.342	**<0.001**
Glycopeptides	61(37.2%)	24(7.3%)	68.293	**<0.001**
Quinolones	80(48.8%)	85(25.9%)	25.646	**<0.001**
3rd/4th generation cephalosporins	30(18.3%)	47(14.3%)	1.301	0.254
1st/2nd generation cephalosporins	23(14.0%)	42(12.8%)	0.142	0.706
Penicillins	6(3.7%)	21(6.4%)	1.587	0.208
β-lactamase inhibitor	103(62.8%)	104(31.7%)	43.383	**<0.001**
Aminoglycosides	7(4.3%)	4(1.2%)	3.359	0.067
Linezolid	19(11.7%)	5(1.5%)	24.044	**<0.001**
Tigecycline	38(23.2%)	10(3%)	50.280	**<0.001**
Daptomycin	3(1.8%)	0(0.0%)	3.396	0.065
Nitroimidazoles	8(4.9%)	13(4.0%)	0.224	0.636
KP detection in other sites Outcome	108(65.9%)	78(23.8%)	82.311	**<0.001**
Death	72(43.9%)	49(14.9%)	49.456	**<0.001**

*KP Klebsiella pneumoniae*, *CRKP* Carbapenem-resistant KP, *CSKP* Carbapenem-susceptible KP, *APACHE* Acute Physiology and Chronic Health Evaluation, *ICU* Intensive Care Unit, *COPD* chronic obstructive pulmonary disease, *CVC* central venous catheter

Multivariable logistic regression showed that KP detection in other sites (*P* = 0.036, OR 1.964), blood purification (*P* = 0.018, OR 3.326), bronchoscopy (*P* = 0.011, OR 5.423), previous surgery (*P* = 0.001, OR 3.084), use of carbapenems (P = 0.001, or 3.395), use of tigecycline (*P* = 0.006, OR 4.595) were independent risk factors for CRKP BSI. Details were shown in Table 4. Hosmer-Lemeshow test showed *P* = 0.177, suggesting that the logistic regression model was reliable.

**Table 4:** Multivariate logistic regression analysis of risk factors for CRKP BSI

Items	*P*	*OR*	95%*CI*
KP detection in other sites	**0.036**	1.964	(1.044, 3.695)
Blood purification	**0.018**	3.326	(1.224, 9.033)
Bronchoscopy	**0.011**	5.423	(1.483, 19.830)
Previous surgery	**0.001**	3.084	(1.572, 6.050)
Use of Carbapenems	**0.001**	3.395	(1.616, 7.134)
Use of Tigecycline	**0.006**	4.595	(1.557, 13.566)

*KP Klebsiella pneumoniae*, *CRKP* Carbapenem-resistant KP, *BSI* bloodstream infections, *OR* Odds ratio, *CI* Confidence interval

### Risk factors of mortality of KP, CRKP and CSKP BSI

The results of univariate analysis of risk factors of mortality in KP, CRKP and CSKP were shown in Table 5. Variables with statistical difference between the death group and survival group in the univariable analysis were included in multivariable logistic regression model.

**Table 5:** Univariate analysis of risk factors associated with death in KP, CRKP and CSKP BSI

Items	KP BSI			CRKP BSI			CSKP BSI		
	Death (*n* = 121)	Survival (*n* = 371)	*P*	Death (*n* = 72)	Survival (*n* = 92)	*P*	Death (*n* = 49)	Survival (*n* = 279)	*P*
Male	72(59.5%)	233(62.0%)	0.506	44(61.1%)	66(71.7%)	0.151	28(57.1%)	167(59.9%)	0.721
Age (years)	63(23)	56(24)	**<0.001**	69(21)	48(29)	**<0.001**	62(17)	59(20)	0.268
previous hospitalization	98(81.0%)	237(63.9%)	**<0.001**	60(83.3%)	61(66.3%)	**0.014**	38(77.6%)	176(63.1%)	0.05
Hospital stay (days)	23(24)	23(23)	0.579	28(27)	36(33)	**0.038**	16(26)	19(18)	0.072
Hospital stay before bacteremia (days) bacteremia (days)	13(26)	5(14)	**<0.001**	14(28)	14(14)	**0.041**	6(24)	3(12)	0.056
APACHE II SCORE	13(7)	8(5)	**<0.001**	14(8)	12(5)	**<0.001**	12(8)	8(4)	**<0.001**
ICU admission	67(55.4%)	107(28.8%)	**<0.001**	53(73.6%)	63(68.5%)	0.473	14(28.6%)	44(15.8%)	**0.03**
Use of systemic steroids	37(30.6%)	77(20.8%)	**0.026**	23(31.9%)	23(25.0%)	0.326	14(28.6%)	54(19.4%)	0.142
Chemotherapy/Radiotherapy	31(25.6%)	71(20.7%)	0.127	17(23.6%)	13(14.1%)	0.119	14(28.6%)	58(20.8%)	0.225
Use of immunosuppressant	33(27.3%)	80(21.6%)	0.195	17(23.6%)	17(18.5%)	0.421	16(32.7%)	63(22.6%)	0.128
Hypertension	57(47.1%)	127(34.2%)	**0.011**	37(51.4%)	35(38.0%)	**0.087**	20(40.8%)	92(33.0%)	0.286
Coronary heart disease	33(27.3%)	71(19.1%)	0.057	25(34.7%)	12(13.0%)	**0.001**	8(16.3%)	59(21.1%)	0.44
Diabetes mellitus	36(29.8%)	122(32.9%)	0.522	21(29.2%)	13(14.1%)	**0.018**	15(30.6%)	109(39.1%)	0.26
COPD	11(9.1%)	12(3.2%)	**0.008**	8(11.1%)	6(6.5%)	0.297	3(6.1%)	6(2.2%)	0.273
Renal diseases	18(14.9%)	24(6.5%)	**0.004**	12(16.7%)	10(10.9%)	0.280	6(12.2%)	14(5.0%)	0.104
Autoimmune diseases	6(5.0%)	11(3.0%)	0.297	4(5.6%)	3(3.3%)	0.740	2(4.1%)	8(2.9%)	0.996
Solid tumor	27(22.3%)	37(10.0%)	**<0.001**	10(13.9%)	9(9.8%)	0.415	17(34.7%)	28(10.0%)	**<0.001**
Hematologic malignancy	21(17.4%)	58(15.6%)	0.654	14(19.4%)	10(10.9%)	0.123	7(14.3%)	48(17.2%)	0.614
Enema	6(5.0%)	17(4.6%)	0.865	6(8.3%)	8(8.7%)	0.934	0	9(3.2%)	0.432
Nasogastric catheter	70(57.9%)	123(33.2%)	**<0.001**	52(72.2%)	65(70.7%)	0.825	18(36.7%)	58(20.8%)	**0.015**
Urinary catheter	74(61.2%)	147(39.6%)	**<0.001**	54(75.0%)	72(78.3%)	0.623	20(40.8%)	75(26.7%)	**0.047**
CVC	95(78.5%)	173(46.6%)	**<0.001**	65(90.3%)	77(83.7%)	0.220	30(61.2%)	96(34.4%)	**<0.001**
Peripheral arterial catheter	16(13.2%)	35(9.4%)	0.235	12(16.7%)	22(23.9%)	0.256	4(8.2%)	13(4.7%)	0.502
Blood purification	19(15.7%)	28(7.5%)	**0.008**	16(22.2%)	20(21.7%)	0.941	3(6.1%)	8(2.9%)	0.461
Tracheal cannula	51(42.1%)	82(22.1%)	**<0.001**	42(58.3%)	52(56.5%)	0.816	9(18.4%)	30(10.8%)	0.129
Tracheostomy	21(17.4%)	35(9.4%)	**0.017**	16(22.2%)	19(20.7%)	0.808	5(10.2%)	16(5.7%)	0.239
Non-invasive ventilation	23(19.0%)	14(3.8%)	**<0.001**	14(19.4%)	8(8.7%)	**0.045**	9(18.4%)	6(2.2%)	**<0.001**
Gastroscopy	3(2.5%)	9(2.4%)	1.000	2(2.8%)	4(4.3%)	0.910	1(2.0%)	5(1.8%)	1.000
Colonoscopy	1(0.8%)	2(0.5%)	1.000	1(1.4%)	1(1.1%)	1.000	0	1(0.4%)	1.000
Bronchoscopy	20(16.5%)	19(5.1%)	**<0.001**	20(27.8%)	15(16.3%)	0.075	0	4(1.4%)	0.890
Sputum suction	58(47.9%)	92(24.8%)	**<0.001**	46(63.9%)	56(60.9%)	0.692	12(24.5%)	36(12.9%)	**0.034**
Thoracentesis	19(15.7%)	27(7.3%)	**0.006**	17(23.6%)	20(21.7%)	0.776	2(4.1%))	7(2.5%)	0.883
Abdominocentesis	11(9.1%)	13(3.5%)	**0.013**	10(13.9%)	9(9.8%)	0.415	1 (2.0%)	4(1.4%)	1.000
Bone marrow puncture	28(23.1%)	54(14.6%)	**0.028**	17(23.6%)	9(9.8%)	**0.016**	11(22.4%)	45(16.1%)	0.278
Lumbar puncture	11(9.1%)	37(10.0%)	0.776	9(12.5%)	15(16.3%)	0.494	2(4.1%)	22(7.9%)	0.519
Previous surgery	45(37.2%)	124(33.4%)	0.449	33(45.8%)	54(58.7%)	0.101	12(24.5%)	70(25.1%)	0.929
Abdominal surgery	36(29.8%)	102(27.5%)	0.631	19(26.4%)	31(33.7%)	0.313	17(34.7%)	71(25.4%)	0.178
Enteral nutrition	47(38.8%)	77(20.8%)	**<0.001**	38(52.8%)	42(45.7%)	0.365	9(18.4%)	35(12.5%)	0.270
Parenteral nutrition Previous use of antibiotics	61(50.4%)	107(28.8%)	**<0.001**	39(54.2%)	56(60.9%)	0.388	22(44.9%)	51(18.3%)	**<0.001**
Any antibiotics	106(87.6%)	281(75.7%)	**0.006**	71(98.6%)	87(94.6%)	0.171	35(71.4%)	194(69.5%)	0.790
Carbapenems	65(53.7%)	75(20.2%)	**<0.001**	51(70.8%)	45(48.9%)	**0.005**	14(28.6%)	30(10.8%)	**0.001**
Glycopeptides	38(31.4%)	47(12.7%)	**<0.001**	30(41.7%)	31(33.7%)	0.295	8(16.3%)	16(5.7%)	**0.02**
Quinolones	52(43.0%)	113(30.5%)	**0.011**	41(56.9%)	39(42.4%)	0.064	11(22.4%)	74(26.5%)	0.548
3rd/4th generation cephalosporins	22(18.2%)	55(14.8%)	0.377	17(23.6%)	13(14.1%)	0.119	5(10.2%)	42(15.1%)	0.372
1st/2nd generation cephalosporins	14(11.6%)	51(13.7%)	0.539	8(11.1%)	15(16.3%)	0.342	6(12.2%)	36(12.9%)	0.899
Penicillins	5(4.1%)	22(5.9%)	0.451	2(2.8%)	4(4.3%)	0.910	3(6.1%)	18(6.5%)	1.000
β-lactamase inhibitor	71(58.7%)	136(36.7%)	**<0.001**	54(75.0%)	49(53.3%)	**0.004**	17(34.7%)	87(31.2%)	0.626
Aminoglycosides	8(6.6%)	3(0.8%)	**0.001**	6(8.3%)	1(1.1%)	0.059	2(4.1%)	1(0.7%)	0.108
Linezolid	14(11.6%)	10(2.7%)	**<0.001**	13(18.1%)	6(6.6%)	**0.024**	1(2.0%)	4(1.4%)	0.557
Tigecycline	26(21.5%)	22(5.9%)	**<0.001**	20(27.8%)	18(19.6%)	0.216	6(12.2%)	4(1.4%)	**<0.001**
Daptomycin	2(1.7%)	1(0.3%)	0.305	2(2.8%)	1(1.1%)	0.830	0	0	**–**
Nitroimidazoles	7(5.8%)	14(3.8%)	0.342	6(8.3%)	2(2.2%)	0.146	1(2.0%)	12(4.3%)	0.726
KP detection in other sites	68(56.2%)	118(31.8%)	**<0.001**	52(72.2%)	56(60.9%)	0.128	16(32.7%)	62(22.2%)	0.114
CRKP	72(59.5%)	92(24.8%)	**<0.001**						

*KP Klebsiella pneumoniae*, *CRKP* Carbapenem-resistant KP, *CSKP* Carbapenem-susceptible KP, *BSI* bloodstream infections, *APACHE* Acute Physiology and Chronic Health Evaluation, *ICU* Intensive Care Unit, *COPD* chronic obstructive pulmonary disease, *CVC* central venous catheter

Multivariate analysis showed that previous hospitalization(*P* = 0.008, OR 2.484), solid tumor(P<0.001, OR 4.960), non-invasive ventilation(*P* = 0.007, OR 4.227) and use of β-lactamase inhibitor(*P* = 0.046, OR 2.007) were independent risk factors for death in KP BSI. And previous hospitalization(*P* = 0.048, OR 2.755), long hospitalization (*P* = 0.003, OR 1.035), bone marrow puncture (*P* = 0.037, OR 3.856), use of β-lactamase inhibitor (*P* = 0.005, OR 3.890) were independent risk factors for mortality in CRKP BSI. Solid tumor (P<0.001, OR 7.068) and non-invasive ventilation (*P* = 0.016, OR 9.778) were independent risk factors for mortality of CSKP BSI. Details were shown in Table 6. The *P* value of Hosmer-Lemeshow tests was 0.885, 0.468 and 0.518, respectively, suggesting that the logistic regression models were sufficiently reliable.

**Table 6:** Multivariate logistic regression analysis of risk factors for mortality of KP, CRKP and CSKP BSI

	Items	*P*	*OR*	95%*CI*
KP BSI	Previous hospitalization	**0.008**	2.484	(1.269, 4.864)
	Solid tumor	**<0.001**	4.960	(2.148, 11.454)
	Non-invasive ventilation	**0.007**	4.227	(1.477, 12.098)
	Use β-lactamase inhibitor	**0.046**	2.007	(1.012, 3.978)
CRKP BSI	Previous hospitalization	**0.048**	2.755	(1.009, 7.541)
	Hospital stay (days)	**0.003**	1.035	(1.011, 1.058)
	Bone marrow puncture	**0.037**	3.856	(1.082, 13.741)
	Use β-lactamase inhibitor	**0.005**	3.890	(1.494, 10.127)
CSKP BSI	Solid tumor	**<0.001**	7.068	(2.683, 18.616)
	Non-invasive ventilation	**0.016**	9.778	(1.529, 62.515)

*KP Klebsiella pneumoniae*, *CRKP* Carbapenem-resistant KP; *CSKP* Carbapenem-susceptible KP, *BSI* bloodstream infection, *OR* Odds ratio, *CI* Confidence interval

## Discussion

This study was focused on CRKP BSI. One of the major findings of our study was the increasing prevalence of KP and CRKP infections, both as a percentage of KP BSI/year and as absolute number. The possible reasons are as follows. In our hospital, the proportion of KP in samples from all sites of the body ranked second in 2014 and 2015(14.0 and 14.2%, respectively), which rose to the first place in 2016–2019(16.1,16.7 and 18.6%, respectively). This may increase its proportion in BSI. Over the past 5 years, the proportion of *Staphylococcus hominis* detected in blood samples was relatively high in 2014 and 2015(22 and 21%), ranking first, then it continued to decline from 2016 to 2019(15, 13 and 11%, respectively). It has been reported that staphylococcus hominis is the main pathogen of blood culture contamination [17]. With the increasing standardization of the blood specimen collection process, the isolation rate of *Staphylococcus hominis* decreased and the proportion of KP BSI increased. In addition, the distribution of CRKP was more and more concentrated in ICU and Hematology Department, where the development of CRKP is much easier, according to the risk factors for CRKP infection based on a meta-analysis [18]. Meanwhile, we found that under the same infection-control measures, the carbapenem resistance among *Acinetobacter Baumannii* and methicillin resistance among *Staphylococcus aureus* isolated from blood samples both decreased while CRKP increased. Combined with his concentrated distribution, we speculate that CRKP BSIs were mostly from endogenous infection. Furthermore, the increasing prevalence of KP and CRKP maybe be related to its special outer membrane proteins, which likely contribute to the integrity and selective impermeability of the cell membrane and also strengthen KP against anionic detergents and certain antibiotics [19].

The proportion of admission to ICU and APACHE II score was higher in CRKP group. Among patients admitted to ICU, 99% (115/116) cases in CRKP group and 86% (50/58) in CSKP group were detected after the admission but not prior to ICU, determining that the CRKP BSI was diagnosed during ICU treatment. So CRKP may not be a factor in ICU admission. Instead, ICU may be the causative factor in CRKP.

At present, the researches about CRKP infection were mostly carried out in ICU patients, which also showed that ICU is a severe area of CRKP infection. ICU has been described as a factory for creating, disseminating, and amplifying antimicrobial resistance [20]. In many previous studies, admission to ICU was regarded as a risk factor for CRKP infection [11, 21–25],though most of these studies that came to this conclusion did not focus on BSI. But ICU admission was not an independent risk factor in the multivariate analysis. This may be due to the presence of other confounding factors. ICU admission itself is just a medical behavior, which does not directly lead to the high prevalence of CRKP. The high prevalence of CRKP in ICU maybe related to the presence of multiple infections in patients, the heavy use of invasive procedures, and the frequent application of high-grade antimicrobials.

In addition to ICU, KP and CRKP BSI were frequent in the hematology and respiratory departments. Most of the inpatients in the Hematology Department are hematologic malignancies. Underlying hematological malignancies, intensive chemotherapy, neutropenia, gastrointestinal mucositis and prolonged hospitalization are all conditions favoring CRKP spread and infections, especially bacteremia [26]. Respiratory tract infection is the most common infection among all infections, followed by urinary tract infection [27, 28]. Antibiotics are widely used in Respiratory Medicine, and even optimal antibiotic use often leads to the development of resistance [29]. Life support, such as tracheal intubation, sputum suction, tracheoscopy and other invasive procedures in critical patients often lead to the damage of airway mucosa, then the bacteria can pass through the barrier into blood flow, which can increase the probability of BSI.

Compared with CSKP group, patients in CRKP group had longer hospitalization time before bacteremia and longer total hospitalization time. It is well known that long hospitalization is a risk factor for the colonization of antibiotic resistant bacteria [30]. Approximately 10% of hospitalizations are complicated by a healthcare-associated infection, and up to 75% of these are due to organisms resistant to first-line antimicrobial therapy [31]. Drug resistant bacteria infection and multi-site infection make conditions more complex and treatment more difficult, which eventually leads to prolonged hospitalization.

The proportion of KP detected in multiple sites in the CRKP group was significantly higher than that in CSKP group. KP detection in other sites was an independent risk factor for CRKP BSI. The concentration of antibiotics in some parts of the body is low, which is insufficient to kill bacteria. Not only that, the inappropriate use of antibiotics may lead to the drug-resistant subpopulations which are present prior to initiation of antimicrobial treatment that may further amplify the resistance [32]. And then the resistant strains of organisms that may become predominant [33]. CRKP in some sites is difficult to be eradicated and eventually spread to the whole body, which will inevitably increase the risk of CRKP BSI. Therefore, for KP BSI with mixed infection in multiple sites, we recommend the antibiotics combination therapy with sufficient tissue penetration and effective concentration to reduce the occurrence of CRKP.

Blood purification, including continuous renal replacement therapy (CRRT), bedside hemofiltration, hemodialysis, plasmapheresis and so on, has not been analyzed in the previous studies. In 2009, a population-based surveillance showed that dialysis patients are more prone to KP bacteremia [34]. In this study, most patients who received blood purification treatment had renal failure, severe acute pancreatitis and shock. They are vulnerable to be infected. And in order to do blood purification, it is requisite to insert the venous catheter first, which belongs to invasive procedures. This may also increase the risk of CRKP infection.

This study suggested that bronchoscopy was an independent risk factor for CRKP BSI. As far as we know, this is the first study to analyze bronchoscopy as a risk factor for CRKP infection. Endoscopy has been considered as a risk factor for CRE transmission for the first time since 2012 [35]. Bronchoscopy is widely used for a variety of diagnostic or therapeutic purposes. Nasopharynx, as one of the common colonization sites of KP [36], is the only way to operate bronchoscopy. This operation may cause mucosal damage in nasopharynx and increase the chance of CRKP entering blood. Furthermore, bronchoscopy may cause the spread of CRKP if it is not well disinfected [35, 37]. But the number of cases in our study is relatively small, which may lead to bias, and further studies are needed to confirm this finding.

Previous surgery (within 1 month) was an independent risk factor for CRKP BSI. Earlier studies have shown that previous surgery was a risk factor for CRKP infection/colonization [21]. Da Silva Kesia Esther et al. suggested that there was a close relationship between surgery and CRKP [38]. Surgery can cause man-made trauma to the human body, increase the chance of bacterial infection, and these patients generally stay in hospital for a longer time, which will also increase the risk of CRKP infection.

The prevalence of drug-resistant bacteria is usually related to the selective pressure caused by the use of antibiotics which changes the patient’s microbiome, causing CRKP to become dominant [25, 26, 33]. Moreover, CRKP can be transmitted through drug-resistant plasmids and it will cause a wider range of CRKP infection [39]. The application of carbapenems can induce the production of acquired *Klebsiella pneumoniae* carbapenemase (KPC) [40], which is one of the main mechanisms of CRKP resistance.

Previous use of tigecycline was an independent risk factor for CRKP BSI. Tigecycline is a glycine antibiotic with activity against many Gram-positive and Gram-negative pathogens [41]. Since the drug resistance rate of bacteria is increasing year by year, tigecycline has become a new choice for severe drug-resistant bacterial infection. The patients exposed to tigecycline included in this study were mostly patients with hematologic malignancies and multiple severe infections caused by more than one kind of drug-resistant bacteria. As a new last line of defense antibiotic, tigecycline was rarely included in risk factor analysis of CRKP infection. However, tigecycline use may cause nausea and vomiting [42], which change the patients microbiome, resulting in CRKP dominance.

It is worth mentioning that as far as we know, this is the first study to consider enemas, one kind of medical procedures, as a potential risk factor, and there was significant difference between the two groups indeed. The gastrointestinal tract is one of the sites of KP colonization [19]. Patients received an enema mostly because of constipation or the preoperative preparation of gastrointestinal surgery. Enemas can cause damage to intestinal mucosa and change the intestinal microbiome. However, the results of this study didn’t not show that an enema was an independent risk factor for CRKP BSI. The number of cases was relatively small and this was a retrospective study, large sample data and prospective study are needed to further determine the relationship between enema and CRKP BSI.

In this study, the mortality of KP BSI was 24.6%, which was lower than 67.6% reported abroad [43]. The mortality of CRKP BSI was 43.9%, which was higher than 14.9% of CSKP BSI. The mortality rates of CRKP infection reported in North America, South America, Europe and Asia were 33.24, 46.71, 50.06 and 44.82% respectively [13]. The mortality rate of CRKP group in our hospital was similar to that in Asia. In the past 5 years, increase in the mortality of KP infections from 14 to 44% as the percentage of resistance increases, implies that resistance is a primary factor in patient mortality, but this was not supported by multivariate analysis. There are also previous studies suggesting that CRKP infection was not a risk factor for the death of KP infection [44, 45]. This finding was supported in the literature review where CSKP were infections from hypervirulent Klebsiella pneumonia (HVKP), which are sensitive to most of the antibiotics except for the natural resistance to ampicillin [46, 47]. But because of high virulence and high pathogenicity, they can also increase the mortality. HVKP has aroused widespread attention. Currently, it has been reported that the mortality of HVKP BSI was 29.2% [48].

Of the patients who died from CSKP, 38.8% (19/49) was the mortality directly related to the infection, others died from liver failure, kidney failure, cardiovascular and cerebrovascular diseases and malignancies. Solid tumor and non-invasive ventilation were independent risk factors for mortality of CSKP BSI. Of the patients who died from CRKP, 61.1% (44/72) directly related to the infection, which was much higher than that of CSKP(*P* = 0.016), others died from severe pancreatitis, hemorrhagic shock, malignancy, organ failure and cerebrovascular disease. The proportion of CSKP in KP BSI was relatively high, and only a small minority died directly from infection, and the death was mainly related to the underlying disease. But once CRKP infection occurs, if it is not well controlled, patients would die from infection.

The long hospitalization and previous hospitalization both reflect the complex conditions and poor therapeutic effects. Bone marrow puncture, as one kind of invasive procedures, is often used in the diagnosis and treatment of patients with hematological diseases. The prognosis of solid tumor patients is generally poor. Most patients are in the middle and late stage when tumor is diagnosed, and the therapeutic effect is hardly satisfactory. Some patients refuse the necessary invasive operations such as tracheal cannula or tracheotomy due to traditional concepts or economic reasons, which may lead to delay of illness or even death.

So, what can we do to reverse this trend? Based on the risk factors for CRKP infection and death, we call for: 1.Screening and determining the microbiome of previous hospitalized patients; 2 Increased resources and more stringent protocols for terminal cleaning of rooms, especially in the ICU, and cleaning of equipment (for example bronchoscopes); 3. Increased attention to and training of physicians regarding antibiotic stewardship; 4. Possible implementing checklists, similar to those of central line-associated bloodstream infection (CLABSI), for procedures such as bone marrow punctures which are performed in the patient’s rooms.

## Conclusion

The prevalence and mortality of CRKP BSI are increasing year by year, suggesting that we need to strengthen the monitoring of the spatiotemporal evolution of CRKP BSI. KP detection in other sites, blood purification, bronchoscopy, previous surgery, use of carbapenems, use of tigecycline were independent risk factors for CRKP BSI. Timely and effective treatment of KP infection in other sites, strengthening the hospital infection control of blood purification, bronchoscopy and recent surgery, enhancing the management of carbapenem and tigecycline and promoting their reasonable application may help prevent and control CRKP BSI. Previous hospitalization, long hospitalization, bone marrow puncture and use of β-lactamase inhibitor were independent risk factors for death in CRKP BSI. To reverse the Upward trend of mortality in KP even CRKP BSI, we appeal to hospitals to:1. Screen and determine the microbiome of previous hospitalized patients; 2. Increase resources and more stringent protocols for terminal cleaning of rooms, especially in the ICU, and cleaning of equipment (for example bronchoscopes); 3. Increase attention to and training of physicians regarding antibiotic stewardship;4. Implement possible checklists for procedures such as bone marrow punctures which are performed in the patient’s rooms.

## Innovation and limitations

Our study focused on KP BSI, analyzed the change of morbidity and mortality of KP and CRKP BSI in our hospital in the past 5 years. We analyzed the department distribution of KP and CRKP for the first time, and included enema, blood purification, bronchoscopy and previous use of tigecycline as possible risk factors in the analysis for the first time. Our study had several limitations: first, this study was a retrospective study; second, this study was a single center study; third, this study only focused on CRKP BSI, lack of analysis and comparison of CRKP infections in other sites and infections caused by other bacteria, even viruses and fungi during this hospitalization; fourth, we used the patients infected with susceptible organisms as controls might generate a selection bias, which may overestimate the effect of carbapenem exposure [49].

## Data Availability

The datasets used and/or analyzed during the current study are available from the corresponding author on reasonable request.

## Abbreviations

KP: *Klebsiella pneumoniae*
CRKP: carbapenem-resistant *Klebsiella pneumoniae*
CSKP: carbapenem-susceptive *Klebsiella pneumoniae*
CRE: carbapenem resistant *Enterobacteriaceae*
CHINET: China Antimicrobial Surveillance Network
BSI: bloodstream infection
KPC: *Klebsiella pneumoniae* carbapenemase
CDC: Centers for Disease Control and Prevention
NHSN: National Healthcare Safety Network
CLSI: Clinical Laboratory Standards Institute
FDA: Food and Drug Administration
APACHE: Acute Physiology and Chronic Health Evaluation
ICU: Intensive Care Unit
COPD: chronic obstructive pulmonary disease
CVC: central venous catheter
OR: odds ratios
CI: confidence intervals
CRRT: continuous renal replacement therapy
HVKP: Hypervirulent *Klebsiella pneumoniae*
CLABSI: Central line-associated bloodstream infection
